# Risk factors for advance stage cardiovascular-renal-metabolic syndrome in patients with early-onset type 2 diabetes mellitus

**DOI:** 10.3389/fendo.2025.1659544

**Published:** 2025-08-27

**Authors:** Yujuan Zhang, Chang Li, Qingyang Leng, Jinghao Pan, Hongli Zhang, Xiaohua Li

**Affiliations:** Department of Endocrinology, Seventh People’s Hospital of Shanghai University of Traditional Chinese Medicine, Shanghai, China

**Keywords:** early-onset type 2 diabetes mellitus, advance stage cardiovascular-renal-metabolic syndrome, risk factors, prediction model, incidence

## Abstract

**Objective:**

To investigate the incidence of advanced cardiovascular-kidney-metabolic (CKM) syndrome and its associated risk factors in patients with early-onset type 2 diabetes mellitus (T2DM).

**Methods:**

This cross-sectional study enrolled 1830 T2DM patients attending Shanghai Seventh People’s Hospital (July 2019–May 2025). Participants were stratified into early-onset (diagnosis age <40 years; n=509) and non-early-onset (n=1321) cohorts. Advanced CKM was defined as stages 3–4 per American Heart Association (AHA) criteria. Comparative analysis, restricted cubic spline (RCS) modeling, binary logistic regression, and receiver operating characteristic (ROC) curves were employed to characterize advanced CKM distribution and determinants.

**Results:**

Advanced CKM incidence was significantly lower in the early-onset group (31.2%, 159/509) versus the non-early-onset group (60.6%, 801/1321) (*P* < 0.001). Among patients with ≤10 years’ disease duration, early-onset individuals exhibited a markedly lower incidence (19.95%, 80/401) compared to non-early-onset counterparts (53.46%) (*P*<0.001). With disease duration >10 years, the early-onset group incidence rose to 68.64% (79/108), converging with the non-early-onset group (76.59%; *P* = 0.08). Binary logistic regression identified independent risk factors for advanced CKM in early-onset T2DM: urine albumin-to-creatinine ratio (UACR; OR = 1.077, 95% CI: 1.046–1.110), blood urea nitrogen (BUN; OR = 1.202, 95% CI: 1.005–1.436), and diabetes duration (OR = 1.102, 95% CI: 1.060–1.145). Protective factors included subcutaneous fat area (OR = 0.995, 95% CI: 0.991–0.999) and antihypertensive medication use (OR = 0.374, 95% CI: 0.199–0.702). The ROC model incorporating these predictors demonstrated an AUC of 0.850 (95% CI: 0.812–0.888) for advanced CKM, with 84.3% sensitivity and 76.8% specificity.

**Conclusion:**

Early-onset T2DM patients exhibit a lower incidence of advanced CKM than non-early-onset individuals, though risk escalates substantially with prolonged diabetes duration. UACR, BUN, and diabetes duration are independent risk factors, while greater subcutaneous fat area and antihypertensive therapy confer protection. The derived prediction model may facilitate early clinical intervention.

## Introduction

Obesity, type 2 diabetes mellitus (T2DM), chronic kidney disease (CKD), and cardiovascular disease (CVD) are pathophysiologically interconnected. The American Heart Association (AHA) designates this complex interplay as cardiovascular-kidney-metabolic (CKM) syndrome (1). CKM pathogenesis involves multifaceted systems and heterogeneous mechanisms. Demographic aging and rising chronic disease prevalence have substantially increased global cardiovascular and renal burdens (2). The CKM syndrome affects more than a quarter of the world’s population (3). Epidemiological studies have shown that between 2011 and 2020, nearly 90% of people in the United States met the CKM stage 1 or higher classification. Even 15% of American adults are classified as advanced CKM (stage 3-4) (4). CKM occurs as a result of hyperglycemia, insulin resistance, elevated activity of the renin-angiotensin-aldosterone system (RAAS), production of late-stage glycosylation end-products, oxidative stress, lipotoxicity, endoplasmic reticulum stress, aberrant calcium handling, mitochondrial dysfunction and impaired energy production, and as well as persistent chronic inflammation and a number of other pathophysiologic mechanisms (5). Large cohort studies have demonstrated that the prevalence of CKM syndromes and their components correlates with the risk of all-cause mortality, CVD mortality, and CKD (6). The prevalence of CKM is also associated with higher rates of CKM and its components. Higher CKM stage is strongly associated with increased mortality and reduced life expectancy (7). The risk of cardiovascular mortality and renal mortality is significantly increased in diabetic patients compared to non-diabetic patients (8).

Early-onset T2DM is defined as T2DM with an age of diagnosis <40 years, where age of diagnosis 18–40 years is defined as early-onset T2DM in adults (9). As the prevalence of diabetes continues to climb, the incidence of early-onset T2DM also continues to grow. During 2016–2022, the average annual incidence of early-onset T2DM among U.S. adults was 3.0 cases per 1000 population. Disproportionately elevated risks were observed among racial minorities (notably African American and Hispanic individuals), socioeconomically disadvantaged groups, and adults with pre-existing cardiometabolic disease (10). Compared to late-onset T2DM, patients with early-onset T2DM have more rapid β-cell failure, a higher prevalence of obesity and other metabolic-related comorbidities, and more difficult glycemic control. Studies have shown that 19.2% of patients with early-onset T2DM had comorbid hypertension at the time of diagnosis, which rose to 46.8% after 10 years of follow-up (11). A meta-analysis of 26 observational cohorts comprising 1.3 million individuals from North America, Europe, and the Asia–Pacific region demonstrated a dose–response relationship: each one-year decrease in age at T2DM diagnosis was associated with a pooled odds ratio of 1.05 (95%CI 1.04–1.06) for microvascular complications and 1.03 (95%CI 1.02–1.04) for macrovascular complications. Macroangiopathic risk increases concomitantly with the duration of T2DM (12).

Nevertheless, current evidence regarding the association between early-onset type 2 diabetes mellitus and the subsequent development of advanced CKM remains limited. The present study therefore aims to estimate the incidence of advanced CKM in individuals with early-onset T2DM, identify its determinants, and develop a predictive model to inform targeted early intervention strategies.

## Methods

### Study design and population

**Figure 1:**
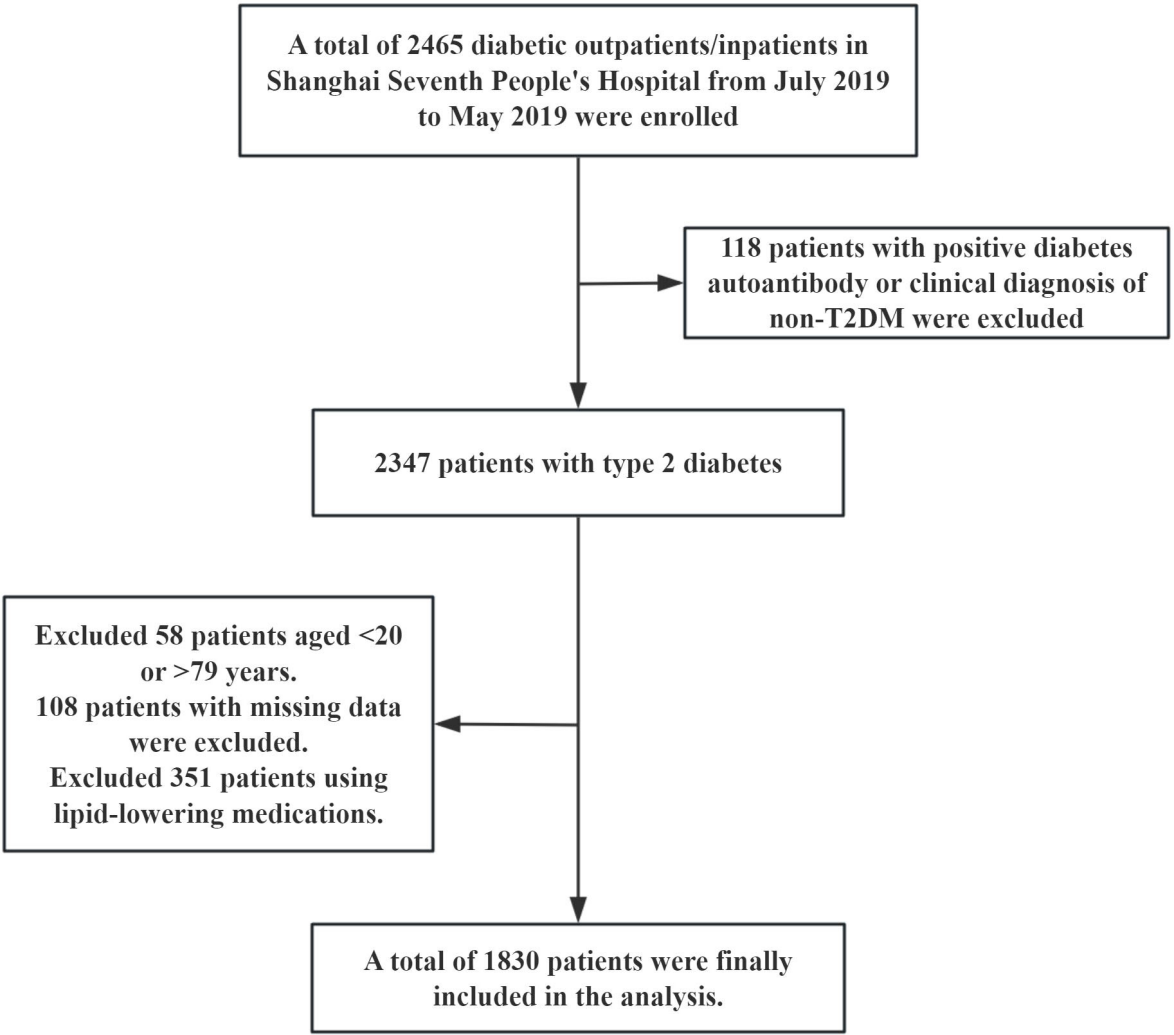
Participant inclusion flowchart.

This study was a cross-sectional observational study. The research subjects were derived from diabetic patients who received treatment in the outpatient or inpatient departments of Shanghai Seventh People’s Hospital from July 2019 to May 2025. A total of 2465 cases were screened and gradually excluded according to the following criteria: (1) A total of 118 cases were diagnosed as non-type 2 diabetes mellitus (non-T2DM) with positive diabetes autoantibodies or clinical diagnosis. (2) 351 patients with previous or current use of lipid-lowering drugs (lipid parameters were included in China-PAR score to exclude the interference of lipid-lowering drugs on lipid levels). (3) There were 58 cases with ages < 20 years old or > 79 years old (the applicable age range for the China-PAR score is 20–79 years old). (4) 108 cases were missing key information. Ultimately, 1830 patients with T2DM were included in the analysis. The specific screening flowchart is shown in **Figure 1**. This study was approved by the Ethics Committee of Shanghai Seventh People’s Hospital (2019-7th-HIRB-010).

### Diagnosis of cardiovascular-renal-metabolic syndrome

According to the recommendations made by the AHA in 2023, CKM syndrome is categorized into 5 stages. Specifically, stage 0 is defined as the absence of any CKM risk factors; stage 1 is defined as overweight, abdominal obesity, or adipose tissue dysfunction (manifested as prediabetes) without the presence of other metabolic risk factors or CKD; stage 2 is defined as the presence of metabolic risk factors or moderate- to high-risk CKD; and stage 3 is defined as a risk equivalent for subclinical CVD, for example, very high-risk CKD or higher 10-year CVD risk as predicted by the Prediction for Atherosclerotic Cardiovascular Disease Risk in China (China-PAR) scoring equation; and Stage 4 is defined as clinical CVD; and, includes coronary heart disease, myocardial infarction, stroke, or peripheral arterial disease (**Supplementary Table 1**). According to the AHA’s scientific statement, CKM stages 3 and 4 are collectively referred to as advanced CKM.The classification of CKD was established based on the Kidney Disease Improving Global Outcomes (KDIGO) criteria, which utilize the estimated glomerular filtration rate (eGFR) and the urinary albumin/creatinine ratio (UACR) (13). The eGFR was calculated using the creatinine formula of the Chronic Kidney Disease Epidemiology Collaboration Without Race or Ethnicity 2021 (14). The China-PAR risk prediction model can be used to predict the risk of atherosclerotic cardiovascular disease for individuals in China over a ten-year period, with good predictive efficacy (http://www.cvdrisk.com.cn) (15, 16).

### Diagnostic criteria for early-onset T2DM

Early-onset T2DM was defined as T2DM patients with onset age < 40 years old. T2DM was diagnosed according to the CDS 2024 guidelines for the diagnosis and treatment of Diabetes (17). Non-T2DM such as type 1 diabetes, monogenic diabetes, and Latent Autoimmune Diabetes in Adults should be excluded.

### Data acquisition

General characteristics such as gender, age, height, weight, Body Mass Index (BMI), waist circumference, hip circumference, systolic blood pressure (SBP), diastolic blood pressure (DBP) were recorded for the participants. Smoking history, alcohol consumption history, past medical history, and co-medication were recorded. Patients were fasted for more than 8 hours, and venous blood was collected the next morning and tested by the hospital testing organization. Alanine Aminotransferase (ALT), Aspartate Aminotransferase (AST), Alkaline Phosphatase (ALP), Gamma-Glutamyl Transferase (GGT), Blood Urea Nitrogen (BUN), Creatinine (Cr), Uric Acid (UA), Total Cholesterol (TC), Triglycerides (TG), High-Density Lipoprotein cholesterol (HDL-c), Low-Density Lipoprotein cholesterol (LDL-c), Fasting Blood Glucose (FBG), postprandial glucose (PBG), Glycated Hemoglobin (HbA1c), Fasting Insulin (FINS), Fasting C-Peptide (FC-P), and other laboratory indicators were collected. The visceral fat area and subcutaneous fat area were measured by experienced nurses using an visceral fat measurement device (HDS-2000 DUALSCAN, OMRON, China).

### Statistical analysis

Statistical analysis was performed using SPSS 26.0 software, and restricted cubic spline plots were drawn using the “rms” program package in R 4.4.2 software. Frequency (N) and percentage (%) were used to describe the categorical variables, and the χ^2^ test was used to compare the groups; if normal distribution was obeyed, $\bar{X}$ ± s was used to describe the numerical variables, and the t test was used to compare the groups. If the distribution was not normal, the median (quartiles) was used to describe the numerical variables, and the Mann-whiteryU test was used for comparison between groups. RCS curves were applied to analyze the dose-response relationship between the incidence of CKM and the age of diabetes onset. Binary logistic regression was used to analyze the factors influencing late CKM in patients with early-onset T2DM. Subject work characteristics (ROC) curves were plotted to analyze the efficacy of influencing factors in predicting late CKM in patients with early-onset T2DM. *P*<0.05 was considered a statistically significant difference. Given that the study period overlapped with the COVID-19 pandemic and the electronic medical records lacked individualized infection records, we adopted time stratification and model robustness tests to reduce potential confounding.

## Results

### Comparison of clinical characteristics between patients with early-onset and late-onset T2DM

This study included 1830 patients with T2DM. Among them, 509 patients with early onset and 1321 patients with non-early onset. Compared with non-early-onset T2DM patients, early-onset T2DM patients had a higher proportion of males, were more likely to have a family history of diabetes mellitus, and had a higher proportion of comorbid fatty liver. Early-onset patients had higher levels of BMI, waist circumference, hip circumference, visceral fat area, subcutaneous fat area, ALT, AST, GGT, UA, TG, HDL-C, LDL-C, FINS, and HOMA-IR (*P*<0.05). Patients with early-onset T2DM had lower rates of postprandial glucose, Cr, BUN, eGFR levels, comorbid hypertension and coronary artery disease, and stroke than those with non-early-onset T2DM, and lower rates of late-onset CKM (*P*<0.05) (**Table 1**). Because the duration of diabetes mellitus was not consistent between the two groups, stratified analyses were performed according to the duration of diabetes mellitus, and consistent results were obtained (**Supplementary Tables 2**, **3**). After adjusting for diabetes duration and other covariates (sex, BMI, HbA_1c_), the incidence of advanced CKM remained significantly lower in the early-onset group than in the late-onset group (OR=0.279, 95%CI 0.220-0.355, *P*<0.001).

**Table 1:** General characteristics of patients with early-onset versus late-onset T2DM.

Variables	Early-onset T2DM (n=509)	Late-onset T2DM (n=1321)	*P*
Gender			<0.001
Male (%)	392 (77)	875 (66.2)	
Female (%)	117 (23)	446 (33.8)	
Age, years	37 (31, 42)	59 (51, 66)	<0.001
Disease duration, years	0.08 (0.00,8.17)	2.83 (0.00,10.17)	0.003
DBP, mmHg	80.00 (70.00,84.50)	80.00 (72.00,85.00)	0.635
SBP, mmHg	130.00 (120.00,133.00)	130.00 (121.00,140.00)	<0.001
Waist circumference, cm	95.80 ± 11.39	91.99 ± 9.72	<0.001
Hip circumference, cm	100.50 (94.50,105.50)	96.50 (91.15,101.50)	<0.001
Visceral fat area, cm^2^	104.25 ± 43.63	94.63 ± 39.12	<0.001
Subcutaneous fat area, cm^2^	208.69 ± 78.97	177.98 ± 57.98	<0.001
Smoking history (%)	169 (33.2)	440 (33.3)	0.966
Drinking history (%)	121 (23.8)	254 (19.2)	0.031
Family history of diabetes (%)	242 (47.5)	492 (37.2)	<0.001
CKM stage			<0.001
Stage 2 (%)	350 (68.8)	520 (39.4)	
Stage 3-4 (%)	159 (31.2)	801 (60.6)	
Hypertension (%)	134 (26.3)	699 (52.9)	<0.001
Coronary heart disease (%)	21 (4.1)	146 (11.1)	<0.001
Stroke (%)	10 (2.0)	56 (4.2)	0.019
Fatty liver disease (%)	291 (57.2)	617 (46.7)	<0.001
ALT, U/L	30.10 (18.35,54.40)	22.15 (15.68,34.73)	<0.001
AST, U/L	20.00 (14.95,33.80)	18.70 (14.60,26.00)	<0.001
ALP, U/L	86.90 (72.95,104.40)	84.85 (69.98,105.53)	0.105
γ-GT, U/L	31.00 (19.00,54.00)	25.00 (17.00,41.00)	<0.001
BUN, mmol/L	4.78 (3.72,5.79)	5.35 (4.41,6.42)	<0.001
Cr, mg/dl	0.64 ± 0.23	0.66 ± 0.21	0.048
UA, mmol/L	340.61 ± 102.04	309.78 ± 84.51	<0.001
FBG, mmol/L	9.30 (7.12,12.07)	8.90 (6.94,11.48)	0.02
PBG, mmol/L	16.81 ± 4.92	17.74 ± 5.36	0.001
HbA_1c_,%	10.43 ± 2.42	9.96 ± 2.33	<0.001
TC, mmol/L	4.74 ± 1.15	4.58 ± 1.16	0.007
TG, mmol/L	1.95 (1.35,3.12)	1.65 (1.17,2.38)	<0.001
LDL-c, mmol/L	3.06 ± 1.01	2.91 ± 1.05	0.008
HDL-c, mmol/L	1.01 (0.89,1.17)	1.10 (0.94,1.29)	<0.001
FINS, pmol/L	60.21 (34.95,105.85)	52.29 (31.98,82.57)	<0.001
F-CP, pmol/L	0.74 (0.52,1.07)	0.71 (0.52,0.94)	0.116
HOMA-IR	4.36 (2.37,7.55)	3.49 (1.99,5.65)	<0.001
HOMA-β	0.38 (0.17,0.74)	0.34 (0.17,0.66)	0.263
eGFR, mL/min/1.73 m²	125.09 (117.54,131.65)	107.11 (99.44,114.95)	<0.001
UACR, mg/mmol	1.47 (0.76,4.25)	1.64 (0.85,3.98)	0.277

SBP, Systolic blood pressure; DBP, diastolic blood pressure; ALT, Alanine Aminotransferase; AST, Aspartate Aminotransferase; ALP, Alkaline Phosphatase; GGT, Gamma-Glutamyl Transferase; BUN, Blood Urea Nitrogen; Cr, Creatinine; UA, Uric Acid; TC, Total Cholesterol; TG, Triglycerides; HDL-c, High-Density Lipoprotein cholesterol; LDL-c, Low-Density Lipoprotein cholesterol; FBG, Fasting Blood Glucose; PBG, postprandial glucose; HbA1c, Glycated Hemoglobin; FINS, Fasting Insulin; FC-P, Fasting C-Peptide.

### Incidence of CKM stages in patients with early-onset T2DM versus patients with late-onset T2DM

**Figure 2:**
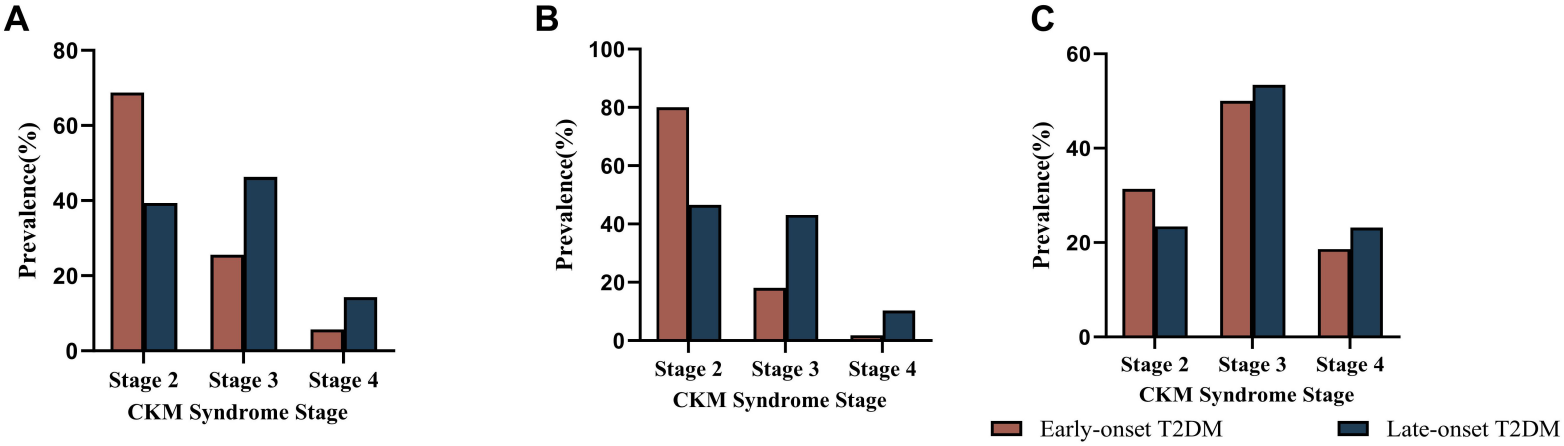
Incidence of CKM by stage in the early-onset and late-onset groups. **(A)** Comparison of the incidence rates of CKM at each stage between early-onset and late-onset T2DM in all patients with type 2 diabetes. **(B)** Comparison of the incidence rates of CKM at each stage between early-onset and late-onset T2DM in patients with type 2 diabetes mellitus within 10 years of the disease course. **(C)** Comparison of the incidence rates of CKM at each stage between early-onset and late-onset T2DM patients with type 2 diabetes mellitus for more than 10 years.

Among the overall included patients, a higher percentage of patients with early-onset T2DM had CKM stage 2 (68.76% vs. 39.36%) and a lower percentage of CKM stage 3 (25.54% vs. 46.33%) and CKM stage 4 (5.7% vs. 14.31%) compared to late-onset T2DM. Further analysis stratified according to diabetes disease duration yielded consistent results. Within 10 years of disease duration, CKM stage 2 accounted for 80.05%, stage 3 18.16% and stage 4 1.79% in early onset T2DM. CKM stage 2 accounted for 46.54% of late-onset T2DM, 43.14% of stage 3, and 10.32% of stage 4. For those with disease duration >10 years, CKM stage 2 was 31.36% in early-onset T2DM, 50.00% in stage 3, and 18.64% in stage4. CKM stage 2 accounted for 23.41% of late-onset T2DM, 53.42% of stage 3, and 23.17% of stage 4 (**Figure 2**). Regression analysis of late CKM incidence with diabetes duration in both groups. The incidence of late CKM increased significantly with the duration of diabetes. This trend was more pronounced in early-onset T2DM (**Supplementary Table 4**).

### Dose-effect analysis between the incidence of late-onset CKM and age of diabetes onset

**Figure 3:**
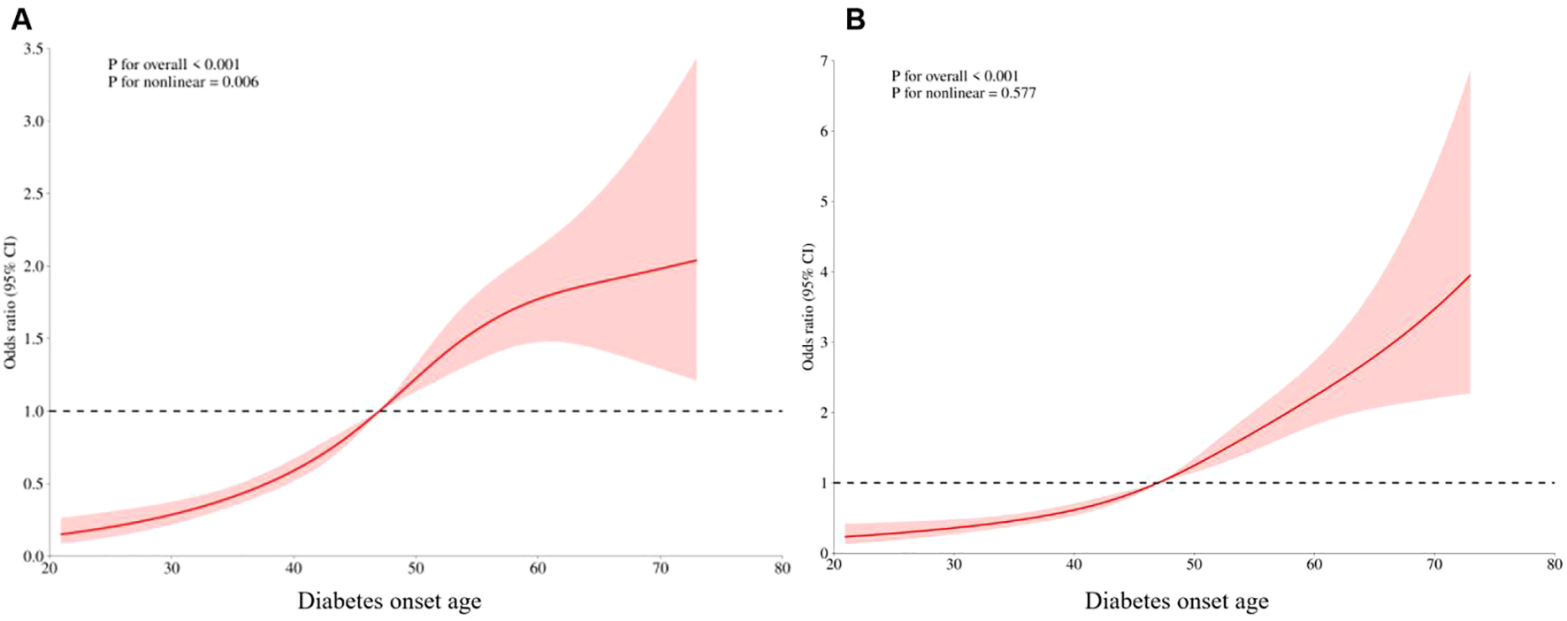
Dose-effect relationship between late CKM incidence and age at diabetes onset. **(A)** Unadjust. **(B)** Adjusting for confounders such as duration of diabetes, gender, and BMI.

RCS curves were used to explore the relationship between the incidence of late-onset CKM and the age of diabetes onset, and 5%, 25%, 75%, and 95% of the age of diabetes onset were set as the 4 nodes of the model. Without adjusting for confounders, a nonlinear dose-effect relationship was observed between the incidence of advanced CKM and age at diabetes onset (*P* for overall<0.001, *P* for nonlinear =0.006). After adjusting for confounders such as duration of diabetes, gender, and BMI, a linear dose-effect relationship was found between the incidence of advanced CKM and age at diabetes onset (*P* for overall<0.001, *P* for nonlinear=0.577) (**Figure 3**).

### General characteristics of patients with advanced CKM in early-onset T2DM compared with patients without advanced CKM

The incidence of late CKM in patients with early-onset T2DM was 31.24% (159/509). Visceral fat area, glycosylated hemoglobin, BMI, waist circumference, hip circumference, subcutaneous fat area, fasting glucose, UACR, ALT, AST, GGT, TG, FINS, F-CP, HOMA-IR, and eGFR were lower in patients with advanced CKM compared to those with non-advanced CKM (*P*<0.05). Cr, HDL-c, SBP, BUN, diabetes duration, hypertension, history of alcohol consumption, fatty liver disease and anti-hypertensive medication utilization rate were higher (*P*<0.05).

### Binary logistic regression analysis of factors influencing late CKM in early-onset T2DM

A one-way regression analysis was performed with whether it was late CKM as the dependent variable (assigned value: yes=1, no=0), and the indicators with *P*<0.05 in **Table 2** were included as independent variables, respectively. Variables with *P*<0.05 were included in multifactorial regression. The results showed that UACR, BUN and duration of diabetes were independent risk factors for the development of late CKM in early-onset T2DM, and subcutaneous fat area and the use of anti-hypertensive drugs were independent protective factors (**Table 3**). Notably, this association was derived from within-subgroup modelling rather than from a between-group comparison. Indeed, baseline UACR did not differ between early- and late-onset groups in **Table 1** (median 1.47 vs 1.64 mg/mmol, *P* = 0.277).

**Table 2:** Comparison of general characteristics of patients with advanced CKM in early-onset T2DM and patients with non-advanced CKM.

Variables	Advanced CKM (n=159)	Non-advanced CKM (n=350)	*P*
Sex			0.918
Male (%)	122 (76.7)	270 (77.1)	
Female (%)	37 (23.3)	80 (22.9)	
BMI, kg/m^2^	25.25 (23.40,28.10)	27.15 (24.68,30.80)	<0.001
Waist circumference, cm	92.50 (88.08,100.50)	96.40 (88.50,103.83)	0.031
Hip circumference, cm	98.50 (92.50,102.80)	100.50 (95.48,107.58)	0.003
DBP, mmHg	80.00 (70.00,85.00)	80.00 (70.00,84.25)	0.654
SBP, mmHg	130.00 (120.00,140.00)	127.00 (120.00,131.00)	0.02
Visceral fat area, cm^2^	96.05 ± 42.28	107.95 ± 43.78	0.005
Subcutaneous fat area, cm^2^	175.55 (136.50,220.00)	205.1 (161,267.175)	<0.001
Hypertension (%)	71 (44.7)	63 (18)	<0.001
Antihypertensive drugs (%)	55 (34.6)	33 (9.4)	<0.001
History of smoking (%)	116 (33.1)	53 (33.3)	0.966
History of alcohol consumption (%)	91 (26)	30 (18.9)	0.08
Family history of diabetes (%)	170 (48.6)	72 (45.3)	0.491
Fatty liver disease (%)	213 (60.9)	78 (49.1)	0.013
ALT, U/L	23.70 (16.28,39.45)	32.05 (20.23,67.53)	<0.001
AST, U/L	17.90 (14.30,26.65)	22 (15.68,36.83)	<0.001
ALP, U/L	84.75 (70.58,99.25)	87.75 (73.85,105.25)	0.167
γ-GT, U/L	25.00 (18.00,41.50)	34.00 (20.00,60.25)	<0.001
BUN, mmol/L	5.40 (4.30,6.69)	4.54 (3.60,5.47)	<0.001
Cr, mg/dl	0.69 ± 0.34	0.61 ± 0.16	<0.001
UA, mmol/L	344.54 ± 108.98	338.81 ± 98.82	0.558
TC, mmol/L	4.69 ± 1.18	4.77 ± 1.13	0.464
TG, mmol/L	1.81 (1.27,2.72)	2.09 (1.39,3.27)	0.024
HDL-c, mmol/L	1.05 (0.91,1.24)	0.99 (0.88,1.13)	0.004
LDL-c, mmol/L	3.01 ± 1.09	3.08 ± 0.97	0.495
FBG, mmol/L	8.93 (6.79,11.65)	9.51 (7.34,12.12)	0.039
PBG, mmol/L	16.35 ± 4.89	17.01 ± 4.93	0.169
HbA_1c_, %	10.03 ± 2.17	10.61 ± 2.51	0.013
FINS, pmol/L	54.99 (33.29,103.35)	63.09 (37.06,110.05)	0.033
F-CP, pmol/L	0.68 (0.45,0.97)	0.76 (0.55,1.08)	0.009
HOMA-IR	3.83 (2.02,6.13)	4.54 (2.58,7.99)	0.008
HOMA-β	0.38 (0.16,0.73)	0.38 (0.18,0.76)	0.273
eGFR, mL/min/1.73 m²	119.05 (109.83,127.29)	126.31 (120.55,133.26)	<0.001
UACR,mg/mmol	2.52 (0.97,25.42)	1.33 (0.69,2.88)	<0.001
Disease duration, years	8.63 (0.56,16.65)	0.00 (0.00,4.08)	<0.001

BMI, Body Mass Index; SBP, Systolic blood pressure; DBP, diastolic blood pressure; ALT, Alanine Aminotransferase; AST, Aspartate Aminotransferase; ALP, Alkaline Phosphatase; GGT, Gamma-Glutamyl Transferase; BUN, Blood Urea Nitrogen; Cr, Creatinine; UA, Uric Acid; TC, Total Cholesterol; TG, Triglycerides; HDL-c, High-Density Lipoprotein cholesterol; LDL-c, Low-Density Lipoprotein cholesterol; FBG, Fasting Blood Glucose; PBG, postprandial glucose; HbA1c, Glycated Hemoglobin; FINS, Fasting Insulin; FC-P, Fasting C-Peptide.

**Table 3:** Binary logistic regression analysis of factors influencing late CKM in early onset T2DM.

Variable	One-way logistic regression analysis		Multifactor logistic regression analysis	
	OR (95% CI)	*P*	OR (95% CI)	*P*
SBP	1.023 (1.006,1.041)	0.007		
BMI	0.919 (0.879,0.96)	<0.001		
Waist circumference	0.984 (0.968,1.001)	0.065		
Hip circumference	0.975 (0.955,0.996)	0.018		
Subcutaneous fat area	0.995 (0.992,0.998)	<0.001	0.995 (0.991,0.999)	0.006
Visceral fat area	0.994 (0.989,0.998)	0.005		
FBG	0.946 (0.894,1.001)	0.054		
HbA_1c_	0.902 (0.832,0.979)	0.013		
ALT	0.986 (0.979,0.992)	<0.001		
AST	0.982 (0.972,0.993)	0.001		
GGT	0.993 (0.988,0.999)	0.014		
BUN	1.526 (1.34,1.737)	<0.001	1.202 (1.005,1.436)	0.044
Cr	5.224 (1.998,13.657)	0.001		
HDL-c	3.156 (1.542,6.46)	0.002		
TG	0.91 (0.822,1.006)	0.066		
FINS	0.997 (0.994,1)	0.032		
F-CP	0.771 (0.51,1.164)	0.216		
HOMA-IR	0.956 (0.921,0.992)	0.017		
eGFR	0.939 (0.923,0.956)	<0.001		
UACR	1.072 (1.044,1.1)	<0.001	1.077 (1.046,1.11)	<0.001
Disease duration	1.152 (1.116,1.188)	<0.001	1.102 (1.06,1.145)	<0.001
Hypertension	0.272 (0.18,0.412)	<0.001		
Antihypertensive drugs	0.197 (0.121,0.32)	<0.001	0.374 (0.199,0.702)	0.002
Fatty liver disease	0.619 (0.424,0.904)	0.013		

BMI, Body Mass Index; SBP, Systolic blood pressure; ALT, Alanine Aminotransferase; AST, Aspartate Aminotransferase; ALP, Alkaline Phosphatase; GGT, Gamma-Glutamyl Transferase; BUN, Blood Urea Nitrogen; Cr, Creatinine; UA, Uric Acid; TG, Triglycerides; HDL-c, High-Density Lipoprotein cholesterol; LDL-c, Low-Density Lipoprotein cholesterol; FBG, Fasting Blood Glucose; HbA1c, Glycated Hemoglobin; FINS, Fasting Insulin; FC-P, Fasting C-Peptide.

### ROC model for risk prediction of late CKM in early-onset T2DM

**Figure 4:**
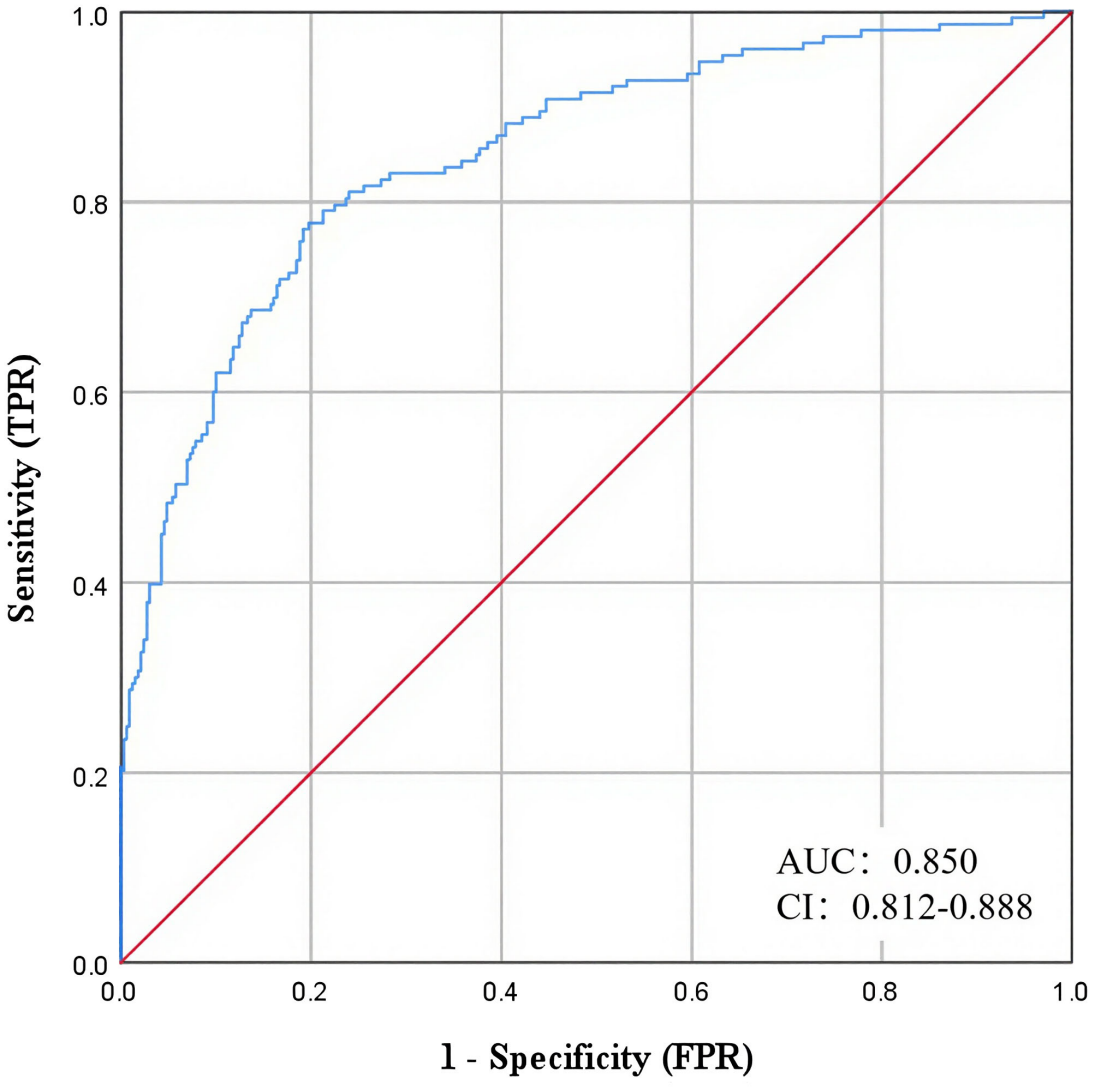
ROC model for risk prediction of advanced CKM in early-onset T2DM.

Based on the above multifactorial binary logistic regression results, a risk prediction model for early-onset T2DM late CKM was constructed (**Figure 4**). The ROC results showed an AUC of 0.850 (95% CI: 0.8122-0.888), suggesting that the model had a good prediction accuracy.

### Sensitivity analysis

The entire cohort was stratified into three subgroups—pre-pandemic (n=36), during-pandemic (n=878), and post-pandemic (n=916)—using January 20, 2020 (the date of the first confirmed COVID-19 case in Shanghai) and January 8, 2023 (the initiation date of “Class B management”) as temporal cut-points. The prevalence of CKM syndrome was compared among the three subgroups (50.00%, 51.59%, and 53.38%, *P*=0.857), with no significant differences observed. Incorporating “year of admission” as a continuous covariate into the multivariable logistic regression model, the interaction term between the year of admission and the China-PAR score was not statistically significant (*P* for interaction=0.627), indicating that temporal trends had limited impact on the primary conclusions.

## Discussion

The global prevalence of cardiovascular-kidney-metabolic (CKM) syndrome continues to climb, affecting more than a quarter of the world’s population. In 2024, approximately 589 million adults aged 20–79 years worldwide had diabetes, and the number of people with diabetes is projected to increase to 853 million globally by 2050 (18). Early-onset type 2 diabetes is also on a significant upward trend. the estimated prevalence of diabetes among people aged 20–39 years grows from 2.9% (63 million) in 2013 to 3.8% (260 million) in 2021 (9). Diabetes significantly exacerbates the risk of cardiac and renal mortality, with cardiovascular mortality elevated 2–4 times in diabetic patients compared to non-diabetic populations. Patients with early-onset type 2 diabetes have more severe metabolic disturbances and are more likely to develop metabolic comorbidities (19). Studies have shown that T2DM patients with diabetes onset age <30 years have a significantly increased risk of myocardial infarction, stroke, and renal failure compared to healthy controls (20).

The present study was a cross-sectional study that included 1830 patients with T2DM, including 509 patients with early-onset T2DM. Consistent with previous studies, metabolic disturbances were more severe in patients with early onset. Higher BMI, visceral fat area, and insulin resistance index were present. However, the present study showed that the incidence of advance CKM syndrome was significantly lower in patients with early-onset T2DM than in the late-onset group. The RCS analysis demonstrated a significant positive correlation between age at diabetes onset and the incidence of late-onset CKM after adjusting for confounders such as diabetes duration, sex, and BMI. This finding is contrary to the conventional wisdom that early-onset type 2 diabetes is associated with a greater risk of cardiovascular disease. This may be closely related to the shorter duration of disease in early-onset patients and the age-dependent protective effect of vascular endothelial function. The median duration of disease was much lower in the early-onset group than in the late-onset group, and the early-onset group had lower systolic blood pressure and a significantly lower prevalence of coronary heart disease and stroke than the late-onset group. Suggesting relatively intact vascular endothelial function and less atherosclerotic plaque conformity. Previous studies have shown that the risk of nonfatal cardiovascular events at 10 years in patients with early-onset T2DM, although lower than that of late-onset patients, inversely exceeds more than 30% at 20 years (21). When the disease duration was >10 years, the risk of late CKM increased steeply in the early-onset group. This nonlinear progression confirms the “metabolic memory” theory. The cumulative duration of hyperglycemic exposure induces mitochondrial oxidative stress, accumulation of advanced glycosylation end products (AGEs) and epigenetic modifications, and ultimately accelerates vascular aging (8).

UACR and BUN as independent risk factors for late CKM in early-onset T2DM explain the pivotal role of the kidney in the CKM syndrome. The early-onset group had significantly better baseline renal function than the late-onset group despite the presence of more severe metabolic disturbances. Multifactorial regression analysis showed a 20.2% increase in the risk of late-onset CKM for every 1 mmol/L increase in BUN (OR=1.202). Elevated BUN signifies an impaired renal tubular urea transporter, leading to urea accumulation. Urea directly activates NLRP3 inflammatory vesicles and stimulates monocytes to secrete IL-1β, driving coronary artery calcification and myocardial fibrosis (3). Albuminuria responds to glomerular basement membrane barrier disruption and increased microvascular permeability.

The present study reveals the protective effect of subcutaneous fat. Visceral fat area was significantly higher in the early-onset group, but subcutaneous fat area was significantly reduced in the advanced CKM group. From an anatomical perspective, visceral adipose tissue (VAT) is mainly located in the mesentery and omentum and is directly drained to the liver through the portal vein system. Compared with subcutaneous areas (SCAT), VAT is more cellular, vascular, and innervative, and contains more inflammatory and immune cells, less preadipocyte differentiation, and a higher proportion of macroadipocytes. Compared with SCAT adipocytes, VAT adipocytes are more metabolically active, more sensitive to fat breakdown, and have stronger insulin resistance (22). Unlike VAT, which releases free fatty acids (FFA) to induce lipotoxicity, SCAT, which enhances skeletal muscle glucose uptake and inhibits hepatic gluconeogenesis through activation of the AMPK pathway. At the same time, adiponectin inhibits macrophage polarization to M1 type and reduces the release of pro-inflammatory factors such as TNF-α and IL-6 (23).

Anti-hypertensive drug use reduced the risk of advanced CKM by 62.6% (OR=0.374). ACEI/ARB analogs block angiotensin II (Ang II) binding to AT1 receptors, inhibit NADPH oxidase activity, and reduce reactive oxygen species (ROS) generation in renal and myocardial tissues, which attenuates collagen deposition (13). RAAS inhibitors improve insulin ARBs can exert antidiabetic effects by activating peroxisome proliferator-activated receptor γ(PPAR-γ), increasing muscle blood flow, up-regulating the expression of glucose transporter proteins in muscle, inhibiting oxidative stress, anti-inflammation, inhibiting fibrosis through inhibition of transforming growth factor β(TGF-β), and enhancing and regulating insulin signaling (24, 25).

Based on the above mechanisms, this study constructed a prediction model to provide a new reference for CKM risk management in early-onset T2DM. Traditional weight loss goals (e.g., BMI < 24 kg/m^2^) may not be applicable to early-onset T2DM. weight management in early-onset T2DM should focus on reducing visceral fat content and promoting fat distribution optimization. Patients with early-onset T2DM should have more stringent glycemic management, with early use of hypoglycemic agents with cardio-renal benefits such as Glucagon-Like Peptide-1 Receptor Agonist (GLP-1RA) and Sodium-Glucose Cotransporter 2 Inhibitor (SGLT2i).

The study period encompasses the entire duration of the COVID-19 pandemic. However, routine electronic health records did not document SARS-CoV-2 nucleic acid or antibody test results, precluding confirmation of individual infection status. Nevertheless, sensitivity analyses revealed consistent prevalence rates of CKM syndrome across the pre-pandemic, during-pandemic, and post-pandemic phases, with no significant interaction between year of admission and the exposure–outcome association. These findings suggest that, at the population level, confounding related to the COVID-19 pandemic had minimal impact on the current conclusions. However, lifestyle changes during the pandemic, such as reduced physical activity and deteriorated dietary quality, may have influenced the cardiorenal–metabolic axis through alternative pathways. Future research should incorporate serological surveys or large-scale linkage of electronic health records to further quantify the independent contribution of COVID-19 to the long-term risk of CKM syndrome in patients with T2DM.

In this study, we focused on the risk of late CKM in early-onset T2DM for the first time and constructed a high predictive efficacy model. The unique disease trajectory of severe metabolic derangement but low initial complications in early-onset T2DM was revealed. The protective effect of subcutaneous fat was revealed, providing a new target for body composition management. However, this study has some shortcomings. First, this study was a single-center cross-sectional study, which did not allow for the establishment of causality, and the inclusion of the population was limited. Second, the study did not record the types of hypoglycemic and antihypertensive medications, which may affect metabolic indicators and CKM outcomes. Future prospective cohorts should integrate electronic prescription databases or pharmacy refill records to accurately quantify medication exposure and disentangle its impact on CKM syndrome risk in patients with T2DM. Finally, we excluded all patients receiving lipid-lowering therapy to minimize confounding of the LDL-C parameter in the China-PAR score by medications. However, this exclusion criterion may have biased the sample towards individuals who did not receive or could not tolerate lipid-lowering therapy, potentially underestimating the CKM syndrome risk in the real-world T2DM population. Future studies should retain information on medication use and employ “lipid-lowering therapy status” as a stratification variable or apply inverse probability weighting to further assess the independent effect of lipid-lowering treatment on CKM outcomes.

## Conclusion

The incidence of late CKM in patients with early-onset T2DM was lower than that in non-early-onset individuals, but the risk increased significantly with the duration of diabetes. UACR, BUN, and duration of diabetes were independent risk factors for late CKM in early-onset T2DM, while subcutaneous fat area and anti-hypertensive therapy were independent protective factors. The prediction model established in this study may provide a reference basis for early intervention.

## Data Availability

The raw data supporting the conclusions of this article will be made available by the authors, without undue reservation.
