# Supplementary material for: Risk factors for advance stage cardiovascular-renal-metabolic syndrome in patients with early-onset type 2 diabetes mellitus
Source: Front Endocrinol (Lausanne). 2025 Aug 27;16:1659544. doi: 10.3389/fendo.2025.1659544 (PMC12420270; doi:10.3389/fendo.2025.1659544)
Supplement: Supplementary file 1 [file Table1.docx]

Supplement Table1. Definition of CKM syndrome stages in this study.

| CKM syndrome stages | Definition |
| --- | --- |
| Stage 0: No CKM risk factors | Individuals with normal BMI and waist circumference, normoglycemia, normotension, a normal lipid profile, and no evidence of CKD or subclinical or clinical CVD |
| Stage 1: Excess or dysfunctional adiposity | Overweight/obesity (BMI ≥ 23 kg/m²), abdominal obesity (waist circumference ≥ 80/90 cm for women/men), or lipodystrophy (fasting blood glucose ≥ 100 ~ 124 mg/dl or HbA1c between 5.7%–6.4%), without other metabolic risk factors or CKD |
| Stage 2: Metabolic risk factors and CKD | Presence of hypertension, diabetes, metabolic syndrome, high triglyceride levels，or any stage of CKD |
| Stage 3: Subclinical CVD in CKM | Subclinical arteriosclerotic CVD or subclinical heart failure in patients with functional metabolic obesity, other metabolic risk factors, or CKD  Subclinical arteriosclerotic CVD: diagnosed through coronary artery calcium scoring  Subclinical heart failure: diagnosed through elevated cardiac biomarkers (NT-proBNP ≥ 125 pg/mL, women/men with sensitive troponin I ≥ 14/22 ng/L, women/men with sensitive troponin T ≥ 10/12 ng/L) or echocardiographic parameters  Subclinical CVD risk equivalent: high-risk CKD and predicted 10-year CVD risk high. |
| Stage 4: Clinical CVD in CKM | Self-reports medication use or seeks medical attention for coronary heart disease, heart failure, or stroke or has CKD with an eGFR of ≤30 mL/min/1.73 m2. |

In this study, the definition of CKM was mainly based on the AHA guidelines. But there are several differences to note: AHA guidelines recommend the use of the PREVENT model for cardiovascular 10-year risk prediction.However, this study mainly included the Chinese population, so the China-PAR model was adopted.

The classification of CKD was established according to the Kidney Disease Improving Global Outcomes (KDIGO) criteria using the estimated glomerular filtration rate (eGFR) and UACR.

CKM: cardiovascular–kidney–metabolic syndrome; BMI: body mass index; CKD: chronic kidney disease; CVD: cardiovascular disease; eGFR: estimated glomerular filtration rate.

Supplement Table 2. Comparison of general characteristics between early-onset T2DM patients and non-early-onset T2DM patients within 10 years of diabetes duration

| Variables | Early-onset T2DM (n=509) | Late-onset T2DM (n=1321) | *P* |
| --- | --- | --- | --- |
| Gender |  |  | <0.001 |
| Male (%) | 316（80.8） | 635（69.7） |  |
| Female (%) | 75（19.2） | 276（30.3） |  |
| Age, years | 35 (30,38) | 55 (48,62) | <0.001 |
| Disease duration, years | 0.00(0.00,3.00) | 0.00(0.00,3.08) | 0.214 |
| DBP, mmHg | 80.00(70.00,84.00) | 80.00(73.00,86.00) | 0.287 |
| SBP, mmHg | 129.00(120.00,132.00) | 130.00(120.00,139.00) | <0.001 |
| Waist  circumference , cm | 97.29±11.38 | 92.79±9.99 | <0.001 |
| Hip circumference,cm | 100.50(95.50,107.50) | 96.75(92.50,102.50) | <0.001 |
| BMI, kg/m2 | 27.40(24.70,30.80) | 25.60(23.40,28.00) | <0.001 |
| Visceral fat area, cm^2^ | 107.00(81.50,140.00) | 97.00(74.00,123.00) | <0.001 |
| Subcutaneous fat area, cm^2^ | 203.00(163.00,268.00) | 178.00(144.75,220.00) | <0.001 |
| Smoking history (%) | 129（33） | 316（34.7） | 0.555 |
| Drinking history (%) | 103（26.3） | 194（21.3） | 0.047 |
| Family history of diabetes (%) | 193（49.4） | 339（37.2) | <0.001 |
| CKM stage |  |  | <0.001 |
| Stage 2 (%) | 313（80.1） | 424（46.5） |  |
| Stage 3-4 (%) | 78（19.9） | 487（53.5） |  |
| Hypertension (%) | 78（19.9） | 430（47.2） | <0.001 |
| Coronary heart disease (%) | 5（1.3） | 72（7.9） | <0.001 |
| Stroke (%) | 2（0.5） | 28（3.1） | 0.005 |
| Fatty liver disease (%) | 242（61.9） | 475（52.1） | 0.001 |
| ALT, U/L | 33.70(20.50,65.50) | 24.10(16.98,40.30) | <0.001 |
| AST, U/L | 22.00(15.40,36.50) | 19.65(15.20,28.53) | 0.001 |
| ALP, U/L | 87.20(73.70,106.00) | 89.05(71.95,108.60) | 0.853 |
| γ-GT, U/L | 35.00(23.00,63.00) | 29.00(19.00,47.25) | <0.001 |
| BUN, mmol/L | 4.49(3.60,5.57) | 5.21(4.22,6.23) | <0.001 |
| Cr, mg/dl | 0.63±0.23 | 0.65±0.21 | 0.149 |
| UA, mmol/L | 347.88±104.87 | 310.79±84.09 | <0.001 |
| FBG, mmol/L | 10.06±3.47 | 9.58±3.32 | 0.017 |
| PBG, mmol/L | 16.81±4.91 | 17.60±5.53 | 0.016 |
| HbA_1c_ ,% | 10.80(9.00,12.20) | 10.40(8.50,11.90) | 0.005 |
| TC, mmol/L | 4.82±1.15 | 4.69±1.16 | 0.052 |
| TG, mmol/L | 2.11(1.42,3.27) | 1.72(1.22,2.53) | <0.001 |
| LDL-c, mmol/L | 3.07(2.52,3.77) | 2.94(2.31,3.68) | 0.066 |
| HDL-c, mmol/L | 0.97(0.86,1.14) | 1.09(0.93,1.27) | <0.001 |
| FINS, pmol/L | 68.02(39.37,121.90) | 54.27(34.03,83.96) | <0.001 |
| F-CP, pmol/L | 0.80(0.59,1.14) | 0.74(0.55,0.99) | 0.009 |
| HOMA-IR | 4.86(2.72,8.39) | 3.71(2.16,5.86) | <0.001 |
| HOMA-β | 0.41(0.20,0.81) | 0.34(0.17,0.66) | 0.010 |
| eGFR, mL/min/1.73 m² | 126.67(121.43,133.24) | 110.37(101.95,117.43) | <0.001 |
| UACR,mg/mmol | 1.36(0.75,3.19) | 1.51(0.83,3.54) | 0.129 |

BMI, Body Mass Index; SBP, Systolic blood pressure; DBP, diastolic blood pressure; ALT, Alanine Aminotransferase; AST, Aspartate Aminotransferase; ALP, Alkaline Phosphatase; GGT, Gamma-Glutamyl Transferase; BUN, Blood Urea Nitrogen; Cr, Creatinine; UA, Uric Acid; TC, Total Cholesterol; TG, Triglycerides; HDL-c, High-Density Lipoprotein cholesterol; LDL-c, Low-Density Lipoprotein cholesterol; FBG, Fasting Blood Glucose; PBG, postprandial glucose; HbA1c, Glycated Hemoglobin; FINS, Fasting Insulin; FC-P, Fasting C-Peptide.

Supplement Table 3. Comparison of general characteristics between early-onset T2DM patients with a diabetes course of more than 10 years and non-early-onset T2DM patients

| Variables. | Early-onset T2DM (n=509) | Late-onset T2DM (n=1321) | *P* |
| --- | --- | --- | --- |
| Gender |  |  | 0.094 |
| Male (%) | 52（68.4） | 176（57.9） |  |
| Female (%) | 24（31.6） | 128（42.1） |  |
| Age, years | 50 (47, 54) | 64 (59, 68) | <0.001 |
| Disease duration, years | 11.17(10.17,15.25) | 11.17(10.17,15.17) | 0.466 |
| DBP, mmHg | 80.00(70.00,84.75) | 80.00(72.00,84.00) | 0.730 |
| SBP, mmHg | 126.00(120.00,136.50) | 130.00(125.00,140.00) | 0.026 |
| Waist  circumference , cm | 90.54±9.78 | 89.92±8.69 | 0.587 |
| Hip circumference,cm | 96.21±7.24 | 94.84±7.26 | 0.141 |
| BMI, kg/m^2^ | 24.58±3.38 | 24.41±3.51 | 0.701 |
| Visceral fat area, cm^2^ | 84.39±35.52 | 85.58±34.25 | 0.789 |
| Subcutaneous fat area, cm^2^ | 156.50(131.25,199.08) | 159.50(127.25,198.00) | 0.467 |
| Smoking history (%) | 25（32.9） | 95（31.3） | 0.783 |
| Family history of diabetes (%) | 33（43.4） | 122（40.1） | 0.602 |
| CKM stage |  |  | 0.002 |
| Stage 2 (%) | 32（42.1） | 74（24.3） |  |
| Stage 3-4 (%) | 44（57.9） | 230（75.7） |  |
| Hypertension (%) | 31（40.8） | 195（64.1） | <0.001 |
| Coronary heart disease (%) | 5（6.6） | 49（16.1） | 0.033 |
| Stroke (%) | 3（3.9） | 18（5.9） | 0.501 |
| Fatty liver disease (%) | 32（42.1） | 115（37.8） | 0.494 |
| ALT, U/L | 22.00(16.33,35.50) | 19.40(13.53,28.53) | 0.057 |
| AST, U/L | 17.85(14.33,24.68) | 17.1(13.73,21.58) | 0.222 |
| ALP, U/L | 84.77±23.20 | 81.75±23.27 | 0.314 |
| γ-GT, U/L | 24.00(16.00,36.25) | 20.00(14.25,28.00) | 0.083 |
| BUN, mmol/L | 5.31(4.65,6.45) | 5.61(4.64,6.92) | 0.092 |
| Cr, mg/dl | 0.60(0.48,0.72) | 0.62(0.52,0.74) | 0.230 |
| UA, mmol/L | 314.10(258.48,372.33) | 303.95(241.33,355.90) | 0.089 |
| FBG, mmol/L | 8.61(6.91,11.66) | 8.28(6.44,11.05) | 0.564 |
| PBG, mmol/L | 17.05±4.90 | 17.89±4.92 | 0.188 |
| HbA_1c_ ,% | 9.89±2.19 | 9.33±2.04 | 0.035 |
| TC, mmol/L | 4.47±1.01 | 4.43±1.15 | 0.803 |
| TG, mmol/L | 1.65(1.22,2.57) | 1.52(1.09,2.32) | 0.194 |
| LDL-c, mmol/L | 2.82±0.94 | 2.71±0.97 | 0.326 |
| HDL-c, mmol/L | 1.05(0.94,1.19) | 1.13(0.93,1.34) | 0.150 |
| FINS, pmol/L | 43.57(23.14,68.37) | 49.75(28.99,77.71) | 0.129 |
| F-CP, pmol/L | 0.56(0.41,0.76) | 0.66(0.44,0.86) | 0.128 |
| HOMA-IR | 2.78(1.74,4.65) | 3.19(1.63,5.26) | 0.201 |
| HOMA-β | 0.23(0.14,0.58) | 0.32(0.17,0.67) | 0.035 |
| eGFR, mL/min/1.73 m² | 117.85(111.46,123.66) | 102.75(97.34,108.51) | <0.001 |
| UACR,mg/mmol | 2.61(1.13,10.46) | 1.85(0.91,5.74) | 0.355 |

BMI, Body Mass Index; SBP, Systolic blood pressure; DBP, diastolic blood pressure; ALT, Alanine Aminotransferase; AST, Aspartate Aminotransferase; ALP, Alkaline Phosphatase; GGT, Gamma-Glutamyl Transferase; BUN, Blood Urea Nitrogen; Cr, Creatinine; UA, Uric Acid; TC, Total Cholesterol; TG, Triglycerides; HDL-c, High-Density Lipoprotein cholesterol; LDL-c, Low-Density Lipoprotein cholesterol; FBG, Fasting Blood Glucose; PBG, postprandial glucose; HbA1c, Glycated Hemoglobin; FINS, Fasting Insulin; FC-P, Fasting C-Peptide.

Supplement Table 4. Correlation analysis of the incidence of advanced CKM and the course of diabetes in the two groups of patients

|  | Variable | β | S.E | t | *P* | β（95%CI） |
| --- | --- | --- | --- | --- | --- | --- |
|  | CKM |  |  |  |  |  |
|  | stage2 |  |  |  |  | 0.00（Reference） |
| Total | stage3-4 | 4.31 | 0.32 | 13.43 | ＜0.001 | 4.31（3.68-4.94） |
| Early-onset T2DM | stage3-4 | 7.70 | 0.65 | 11.80 | ＜0.001 | 7.70(6.42-8.98) |
| Late-onset T2DM | stage3-4 | 3.33 | 0.38 | 8.72 | ＜0.001 | 3.33（2.58-4.08） |
